# Scraps

**Published:** 1891-12

**Authors:** 


### SCRAPS

PICKED UP BY THE ASSISTANT-EDITOR.

---

**Clifton Spa.**—As there is now an attempt to restore Clifton and the Hotwells to something of its former reputation as a health-resort, it will be appropriate to quote some lines from "Clifton," a poem by that much neglected son of Bristol, Chatterton:—

Clifton, sweet village! now demands the lay,
The loved retreat of all the rich and gay;
The darling spot which pining maidens seek,
To give health's roses to the pallid cheek.
Warm from its font the holy water pours,
And lures the sick to Clifton's neighbouring bowers.
Let bright Hygeia her glad reign resume,
And o'er each sickly form renew her bloom.
Me, whom no fell disease this hour compels
To visit Bristol's celebrated wells,
Far other motives prompt my eager view;
My heart can here its favourite bent pursue;
Here can I gaze, and pause, and muse between,
And draw some moral truth from every scene.

The yellow Avon, creeping at my side,
In sullen billows rolls a muddy tide;
No sportive Naiads on her streams are seen,
No cheerful pastimes deck the gloomy scene;
Fixed in a stupor by the cheerless plain,
For fairy flights the fancy toils in vain:
For though her waves by commerce richly blest,
Roll to her shores the treasures of the west,
Though her broad banks trade's busy aspect wears,
She seems unconscious of the wealth she bears.
Near to her banks, and under Brandon's hill,
There wanders Jacob's ever-murmuring rill,
That, pouring forth a never-failing stream,
To the dim eye restores the steady beam.

Chatterton, although he had at one time a desire to enter the medical profession, freely satirised the local doctors. Much of the satire is in his poem "The Exhibition," which for very good reasons has never been printed in full. Mr. Greig Smith in his *Abdominal Surgery*, 2nd Ed. p. 631, quotes from Chatterton some lines on one of the Bristol surgeons, and refers to "an unpublished, and unpublishable, poem now in the library of the Bristol Royal Infirmary."

**Household Science.**—A contemporary publishes the following amusing results of a late examination of girls in "Household Science." Their ages ranged from twelve to eighteen. They had entered from schools of all kinds, Government and private. Some questions concerning food and processes of cookery elicited the following answers: "Potatoes, when done, should be allowed to sit with a towel over them." "Tea, coffee, and cocoa are usually adulterated with beer, ale, and brandy." "Brown bread is more bulky and more spongy, and helps to distend the stomach better than white bread. Therefore it is more wholesome." "Brown bread forms a crust on the stomach." "Carbonaceous foods perform heat and make people fat; nitrogenous foods perform force." "Get a shoulder of mutton, trim it from the neck neatly, leave an inch or two of bare bone for a handle, add a pinch of pepper and salt; then put it on the pan and six or seven minutes will cook it." "Begin to prepare cutlets by paring the chops of the sheep." "Do not throw away the small crags of mutton." "Have your frying-pan ruthlessly clean." "A few hours should be sufficient to cook a cutlet." "Cutlets should be boiled in cold water, and served cold with some parsley and warm potatoes." "Brown bread irritates the intestates." "Each cutlet should be an inch long." "Brown bread ruffles the inside of the gullet." Some of the suggestions given for the treatment of simple ailments were as follows: "Poison should be taken out with a tweezers." "If you want to keep a room healthy, sit in it with a bowl of lime-water." "In case of a convulsive fit, speak to the patient sharply." "Place the person who has fainted in a lying posture, keep him quiet, and wait till he comes round." "If you want to know your temperature, put a barometer into your mouth." "The heat of the body is produced by putting a thermometer in your mouth." A girl was told to draw out a classification of foods according to the work they perform. She drew diagrams of a cow and a sheep, and divided them into joints of butcher's meat. The following are the kaleidoscopic forms into which the term "warp and woof" was shaken: "the warft and the werft," "the waft and the wept," "the wift and the weft," "the werp and the warp," "the weave and the worp," "the worp and the wemp," "the theft and the weft," and "the yelp and the welp."

**Medical Advertising.**—A subscriber writes to me as follows:—"I have the originals of these two handbills, the second of which was circulated as late as 1857. The advertising in those days was more blunt, but probably not more effectual than the samples we have around us now."

NOTICE.

SURGEON TODD is going to set up in Pointspass a druggist's shop, nearly at the foot of Newry Road, upon the right hand of the street, and he is going to do the Publick at large the favour to inform them of his profession, he professes Surgery, Midwifery and Druggist to all those that will do themselves the favour to call upon him in time of need, and in like manner with the help of God he proposes to cure all the Disorders that the Human Body is liable to; and as for his Drugs, you shall have them at the most reasonable terms, and those that will come to his shop for medicines shall have directions how to use them and his advice gratis free.

remains yours,

Surgeon TODD.

April the 23rd, 1807.

---

TO THIS PLACE HAS COME
MR. MOSES EVANS,
SURGEON.
*(Late of Bristol Infirmary.)*

MR. M. EVANS returns his grateful acknowledgments to the candid public in general, and begs leave to inform them that he intends visiting this circuit four times a year. It is well known to all acquainted with him, that close application to business and upwards of 13 years practice has raised him to a degree of eminence in his profession. But he is not wishful to pass voluminous encomiums on his skill, as it may be satisfactorily proved by all who desire to know the nature and causes of their diseases, when this Bill will be called for to-morrow.

---

FIVE POUNDS REWARD. CAUTION!

In consequence of many unprincipled persons coming around this neighbourhood, representing themselves as being connected with Mr. Moses Evans, this is to give notice to the public, that he is the only person of that name visiting the circuit, who has received a Medical education; therefore any person who will give such information as will lead to their conviction, shall receive the above reward.

*Cardiff, October* 17, 1857. W. Jones, Printer, Bridgend House, Pontypridd.

